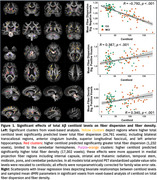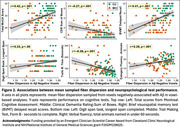# Centiloid levels predict altered brain microstructure in adults without dementia

**DOI:** 10.1002/alz70862_109910

**Published:** 2025-12-23

**Authors:** Andrew R. Bender, Pavithran Pattiam Giriprakash, Zhengshi Yang, Dietmar Cordes, Jessica ZK Caldwell

**Affiliations:** ^1^ Cleveland Clinic Lou Ruvo Center for Brain Health, Las Vegas, NV USA

## Abstract

**Background:**

Alzheimer’s disease is associated with altered brain microstructure, measurable via diffusion‐weighted magnetic resonance imaging (dMRI). Extant findings suggest later‐developing, smaller‐caliber axons are particularly vulnerable to early‐stage Alzheimer’s neuropathologies; we hypothesized such changes are observable via dMRI measures of fiber density (FD) and dispersion. We investigated whether elevated amyloid‐β (Aβ) predicts differences in FD and fiber dispersion in older adults without dementia, and the sensitivity of Aβ‐associated microstructural parameters to cognitive function.

**Method:**

Data from 122 participants (45% F; 7.4% NHW) enrolled in the Center for Neurodegeneration and Translational Neuroscience included those characterized as cognitively unimpaired (*n* = 58) or consensus diagnosed with amnestic mild cognitive impairment (*n* = 64). Participants underwent multi‐shell dMRI and amyloid PET neuroimaging; all completed standardized neuropsychological tests of visual reproduction, executive function, working memory, and semantic fluency. We calculated standardized uptake value ratio from PET data, rescaled to centiloids. DMRI processing leveraged MRtrix3 to model total FD and fiber dispersion as voxelwise scalars. Separate mass‐univariate voxel‐based analyses assessed total centiloid level predicting differences in total FD and dispersion, while accounting for age, sex, handedness, and head size; all results are nonparametrically corrected for familywise error rate.

**Result:**

Higher Aβ predicted widely *reduced* fiber dispersion in transcallosal regions, anterior cingulum bundle, superior longitudinal fasciculi, and left anterior hippocampus, but predicted *increased* dispersion in cerebellar hemispheres (Figure 1). Post hoc regressions of mean fiber dispersion in Aβ‐negative voxels showed remarkably strong associations with centiloid level (*r* = –0.78, *p*<.001, adjusted‐*R^2^* = 0.64). In contrast, Aβ positively predicted FD in striatal, thalamic, midbrain, and cerebellar projection fibers, and in corpus callosum‐genu (*r* = 0.34, *p*<.001). Regressions of cognitive performance showed mean fiber dispersion significantly predicted CDR sum‐of‐boxes, delayed recall, verbal fluency, working memory, and executive function (Figure 2), controlling for age, sex, and education; associations between FD and behavior were attenuated by age and sex.

**Conclusion:**

Aβ neuropathology is associated with altered white matter organization in community‐dwelling adults without dementia. Fiber dispersion is a novel dMRI measure with exceptional correspondence to Aβ‐PET centiloid levels and cognition, independent of age and sex. Modeling fiber density and dispersion afford complementary insights about microstructural changes associated with Alzheimer’s pathology and cognitive function.